# Factors Influencing the Long-Term Survival and Success of Endodontically Treated and Retreated Teeth: An Ambispective Study at an Educational Hospital

**DOI:** 10.3390/jcm14217826

**Published:** 2025-11-04

**Authors:** Reem Barakat, Rahaf Almohareb, Ghaliah Alsawah, Hadeel Busuhail, Shahad A. Alshihri, Ghadah T. Alrashid, Ghadeer Y. Alotaibi, Mamata Hebbal

**Affiliations:** 1 Dental Clinics Department, King Abdullah bin Abdulaziz University Hospital, Princess Nourah Bint Abdulrahman University, Riyadh 11671, Saudi Arabia; rmbarakat@pnu.edu.sa; 2 Department of Clinical Dental Sciences, College of Dentistry, Princess Nourah Bint Abdulrahman University, Riyadh 11671, Saudi Arabia; raalmohareb@pnu.edu.sa (R.A.); hbasuhail99@gmail.com (H.B.); shahadahmed847@gmail.com (S.A.A.); alghada456@gmail.com (G.T.A.); ghadeer.alotaibii4@gmail.com (G.Y.A.); 3 Department of Preventive Dental Sciences, College of Dentistry, Princess Nourah Bint Abdulrahman University, Riyadh 11671, Saudi Arabia; mihebbal@pnu.edu.sa

**Keywords:** endodontically treated teeth, root canal treatment, coronal restoration, survival analysis, treatment success

## Abstract

**Background**: This ambispective study evaluated the prognostic factors for long-term survival and success of endodontically treated teeth (ETT) in patients treated at an educational hospital. **Methods**: Patients who received root canal treatment (RCT) at Princess Nourah bint Abdulrahman University (2018–2023) were included if the following criteria were met: follow-up time of a minimum of 12 months was available, and clear pre- and post-treatment periapical radiographs. Two calibrated examiners assessed RCT quality along with pre-treatment and follow-up periapical index (PAI) scores. Coronal restorations were evaluated for quality, type, and timing. Data on oral hygiene, smoking, systemic health, periodontal status, and occlusal factors were collected. Tooth survival was defined as a functional, asymptomatic tooth, while success required a clinically asymptomatic tooth with a PAI score ≤ 2. Multivariate regression analysis was performed to assess the influence of the collected variables on tooth survival and success. Statistical significance was set at *p* < 0.05. **Results**: A total of 461 ETT from 242 patients were analyzed. The survival rate was 89%, while 81% met the treatment success criteria. Most patients were female (65%), and only 8.9% had a healthy periodontium. Multivariate analysis identified preoperative PAI score, obturation density, and coronal restoration quality as significant predictors of survival and success (Nagelkerke R^2^ = 0.338). **Conclusions**: ETT demonstrated high survival and success rates. Preoperative PAI score, obturation density, and coronal restoration quality were key predictors of long-term treatment outcomes, highlighting the importance of comprehensive pre-treatment assessment and high-quality endodontic and restorative procedures.

## 1. Introduction

The primary objective of root canal treatment (RCT) is to prevent or resolve periapical disease, and thereby preserve the natural tooth. However, directly assessing the root canal system and periapical tissues to determine whether infection has been successfully eradicated presents challenges due to their inaccessibility. Consequently, various criteria have been proposed to evaluate RCT outcomes. Historically, outcomes have been classified as either success versus failure or healing versus disease, typically based on a combination of clinical and radiographic findings. Radiographic success criteria, however, vary depending on the definitions applied.

“Loose” criteria consider a reduction in periapical lesion size as indicative of success, whereas “strict” criteria require complete radiographic resolution of apical periodontitis [1]. A meta-regression analysis suggested that applying stricter criteria may reduce reported success rates by approximately 10.5% [2]. Using two-dimensional radiographic evaluation, the success of initial RCT ranges from 82% under strict criteria to 92.6% under loose criteria [1]. When cone-beam computed tomography (CBCT) is used, the variation between the two definitions becomes even more pronounced ranging from 36% to 88% [3].

Friedman and Mor (2004) introduced the concept of functional retention, defined as a tooth remaining in the mouth without causing discomfort to the patient [4]. From this perspective, root canal treatment (RCT) can be considered successful if the tooth remains functional and symptom-free, regardless of its radiographic appearance—a criterion commonly referred to as survival [5]. Understanding the factors associated with tooth survival can guide treatment decisions and preventive strategies, thereby enhancing patient care [6]. However, the potential health implications of prioritizing functional retention remain uncertain [7,8]. Tooth survival is a key metric for assessing the success of dental treatments, particularly RCT, which aims to prevent tooth extraction [9]. While some studies suggest that the quality of RCT alone determines its outcome [10,11], others indicate that multiple factors play a role, including patient characteristics (age, health status, and gender) and operator skill and experience. Tooth-specific factors—including preoperative diagnosis, tooth position, follow-up time, and type of restoration—also significantly influence outcomes [1].

The preoperative pulpal diagnosis, particularly whether the tooth is undergoing initial treatment or requires secondary RCT (retreatment), influences the rate of success. Multiple studies have reported lower success rates for secondary RCT due to associated technical challenges such as obstructed or altered canal anatomy and/or the presence of long-standing resistant infection [12,13,14]. Nevertheless, some evidence suggests that the difference between initial and secondary RCT is not significant [15,16,17].

The placement and type of coronal restoration are critical clinical factors influencing the survival of endodontically treated teeth (ETT) [18,19,20]. Some studies indicate that the quality of the coronal restoration may have a more substantial impact on treatment outcomes than the quality of root canal filling [21,22,23,24]. Conversely, other studies suggest that the presence of pre-treatment apical periodontitis may influence restoration choices, potentially introducing bias. These studies found no significant association between the presence of a permanent restoration and RCT outcomes [15,25,26].

Therefore, this retrospective study aims to analyze records of teeth that underwent initial or secondary non-surgical RCT in an educational hospital setting, evaluating various predictive factors—in relation to the survival and success outcomes of endodontically treated teeth.

## 2. Materials and Methods

### 2.1. Ethical Approval

This retrospective study received ethical approval from the Institutional Review Board at Princess Nourah bint Abdulrahman University (PNU) (PNU IRB #23-0564). This research adhered to the principles of the Declaration of Helsinki.

### 2.2. Study Population

Patients who underwent non-surgical RCT at the PNU Dental Clinics between April 2018 and February 2023, after obtaining informed consent, as documented in the clinics’ AxiUm electronic health records (Exan 7.02, Henry Schein, Melville, NV, USA), were considered for inclusion. Eligible patients were required to be at least 18 years old, have comprehensive pre-treatment data recorded in AxiUm—including dental and medical history, clinical signs and symptoms, endodontic diagnosis, and test results—and possess clear pre- and post-operative periapical radiographs. Additionally, at least 12 months had to have elapsed since the completion of their RCT [1].

A total of 1500 patients who met the inclusion criteria were contacted and offered recall appointments. During these appointments, the study’s purpose was explained, and patients were informed that participation was voluntary and would not affect their ongoing treatment at the PNU Dental Clinics.

Case difficulty was assessed using the American Association of Endodontists (AAE) Case Difficulty Assessment and distributed among undergraduate dental students, interns, endodontic board residents, and endodontists. All students, interns, and residents worked under the close supervision of an endodontist with a maximum ratio of 4:1, adhering to standard clinical protocols. If a mishap occurred in the undergraduate clinics, the case was promptly referred to the graduate or endodontist specialist clinics for management. All non-surgical RCT procedures at the PNU Dental Clinics were performed under rubber dam isolation in compliance with clinical regulations. Canal preparation was carried out using a combination of manual and rotary files, with irrigation using 5.25% sodium hypochlorite delivered through 30-gauge Luer-lock Endo irrigation needles. The canals were prepared to a minimum apical size of 30. EDTA was applied either as a lubricant gel and/or as a final irrigant. When indicated, calcium hydroxide was placed as an intracanal medicament for a minimum of one week between treatment visits. The standard obturation protocol in the clinics involved using gutta-percha cones with a taper of 2% or greater in combination with AH Plus sealer. During retreatment, gutta-percha removal was achieved using gutta-percha solvents in combination with hand and rotary retreatment files.

### 2.3. Clinical and Radiographic Assessments

A total of 242 patients attended the recall visits, representing a recall rate of 16.1%. During the recall visits, both clinical and radiographic assessments were performed, including periapical and bitewing radiographs. Two calibrated examiners conducted comprehensive evaluations to assess the quality of RCT and pre-treatment and follow-up periapical index (PAI) scores, employing a validated five-point scale [27]. Teeth were categorized based on their PAI scores: a score of 1 or 2 indicated a healthy condition, while a score of 3 to 5 signified periapical disease. For multi-rooted teeth, the classification was determined by the root with the highest (worst) PAI score.

Digital periapical radiographs were reviewed using Mipax 3.2 software (Microtek, Taiwan). All images were examined at 1.5× magnification on a 21-inch LCD monitor with a resolution of 1920 × 1200 pixels at 60 Hz in a darkened room with consistent ambient lighting. Contrast and brightness adjustments were applied to optimize image visualization.

The evaluation criteria for RCT quality focused on the length and density of the root canal filling. An adequate root canal filling was defined as one that terminated at or within 2 mm of the radiographic apex. An inadequate filling either extended beyond the apex or fell more than 2 mm short. Filling density was considered adequate when no visible voids were present within the material or between the filling and canal walls [28,29].

Coronal restoration quality was assessed clinically and radiographically by a single restorative dentist with more than five years of experience. A restoration was classified as adequate if it was intact, demonstrated proper marginal adaptation, and was free from fractures, cracks, or recurrent caries. Inadequate restorations were classified as inadequate if they exhibited visible overhangs, fractures, cracks, poor marginal adaptation, or recurrent caries.

Clinical examination also assessed tenderness to palpation and percussion; the presence of pain, swelling, or sinus tracts; tooth mobility; occlusal status (including the type of coronal restoration, presence and type of post, and timing of the most recent restoration; and the operator who performed the procedure. Additional data on patients’ oral hygiene practices, oral habits, smoking status, medical conditions, and periodontal status were recorded.

All data were documented and coded in an Excel spreadsheet, with each tooth treated as a separate unit. Treatment was considered successful if the tooth was asymptomatic and the PAI score at follow-up was either 1 or 2, indicating the absence of disease. Tooth survival was defined as the presence of asymptomatic teeth regardless of radiographic findings.

### 2.4. Reliability of Measurements

Inter-rater and intra-rater reliability for PAI scoring and RCT quality evaluation were assessed using Cohen’s kappa statistics. Calibration for PAI scoring achieved a kappa value of 0.69, indicating acceptable agreement, while intra-observer agreement reached 0.85, demonstrating excellent consistency. Inter- and intra-operator agreement for RCT quality evaluation ranged from 0.836 to 1.0, indicating excellent agreement.

### 2.5. Data Management and Statistical Analysis

The collected data were analyzed using JMP^®^ Pro (version 18.0.2, JMP Student Edition). Descriptive statistics were used to summarize the data, with categorical variables expressed as frequencies and percentages. The Chi-square test was applied to assess associations among study variables. Multiple logistic regression analysis was conducted to evaluate the relationship between study variables and both survival and success rates of RCT. Multicollinearity was addressed by including only one variable from each set of closely related predictors, selected based on expert opinion or supporting evidence. Statistical significance was set at *p* ≤ 0.05.

## 3. Results

**Figure 1:**
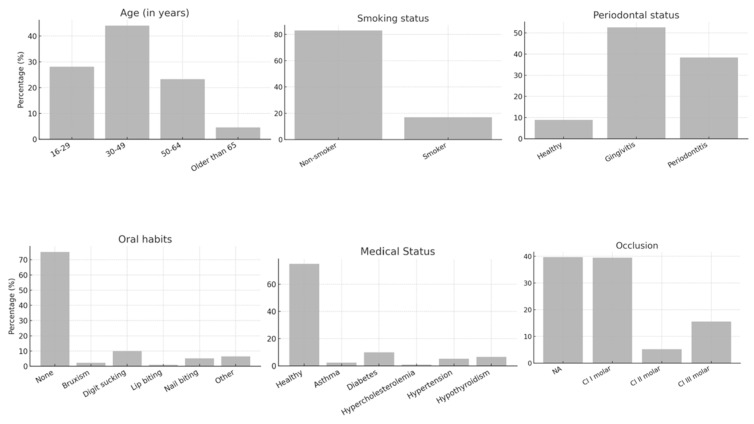
Distribution of demographic and patient related variable in the study sample.

**Figure 2:**
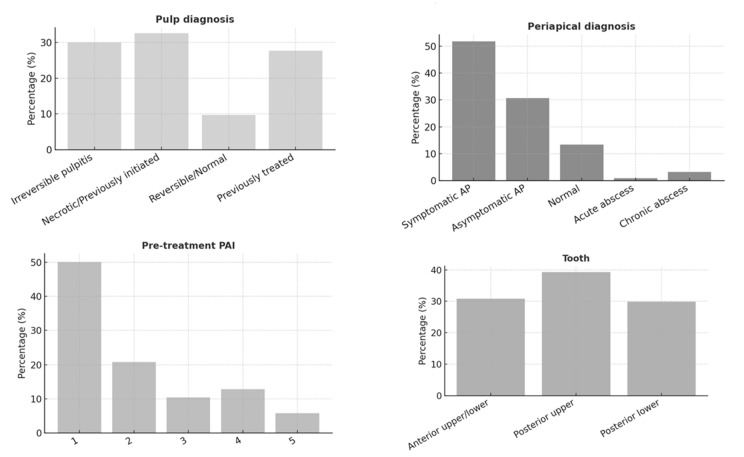
Frequency distribution of tooth-related factors including tooth type along with pre-opt pulpal and periapical diagnosis. AP: apical periodontitis.

**Figure 3:**
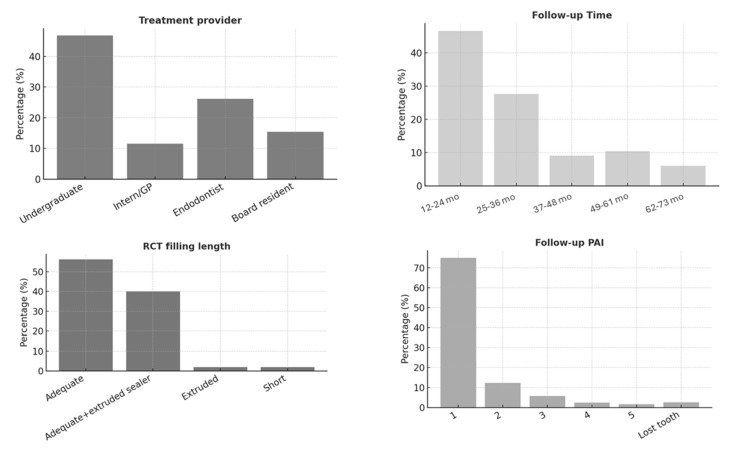
Frequency distribution of treatment-related factors in the study sample including type of endodontic treatment provider, follow-up time and the Periapical Index (PAI) score at follow-up.

**Figure 4:**
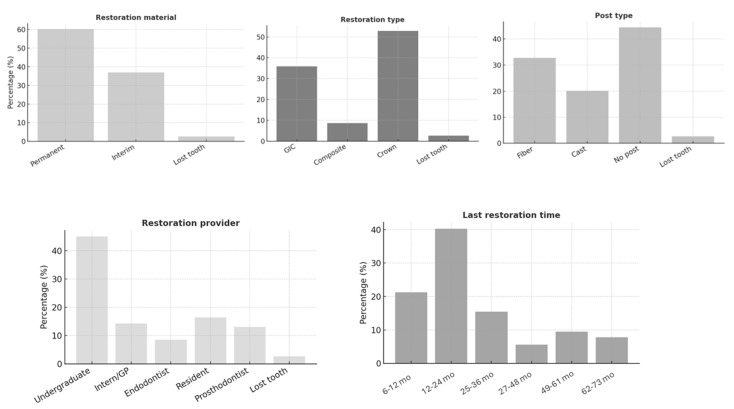
Restoration-related characteristics of the study sample presented as frequency distributions including restoration material type, presence of post and elapsed time since restoration placement.

The study included 461 endodontically treated teeth (ETT) from 242 patients. The majority of patients (44.3%) were aged between 30 and 49 years, with 65% being female. Most participants (75%) were medically healthy; however, only 8.9% had a healthy periodontium, and 53.6% exhibited fair oral hygiene (Figure 1). Figure 2, Figure 3 and Figure 4 present the distribution of key variables in the study sample, including tooth-related factors such as pre-treatment endodontic diagnosis of the tooth, root canal treatment-related factors encompassing treatment provider, follow-up time and factors related to coronal coverage of the ETT. Among patients who attended the follow-up visit, treatment had been completed 12–24 months (mean: 16. prior in nearly half of the cases (46.6%) (Figure 3). Filling density was deemed adequate in 98% of cases, with a low prevalence of short or overextended fillings (3.9%). Provider experience was strongly correlated with treatment success (*p* < 0.001), as was the preoperative PAI score (*p* < 0.001).

ETT demonstrated a high survival rate of 89.1%. Among demographic factors, age (*p* = 0.004) and oral habits (*p* = 0.04) were significant predictors. Additionally, patients’ medical status (*p* = 0.003), periodontal status (*p* = 0.013), and oral hygiene (*p* = 0.022) were significantly associated with tooth survival.

Treatment-related factors—including follow-up time (*p* = 0.001), treatment provider (*p* = 0.038), quality of endodontic filling (*p* < 0.001), coronal restoration quality (*p* < 0.001), type of restorative material (*p* < 0.001), and post use/type (*p* < 0.001)—also showed significant associations with tooth survival.

Bivariate analysis revealed that the density of the RCT filling, as well as the type and quality of the coronal restoration, were significant prognostic factors for tooth survival. The density of endodontic filling showed an odds ratio (OR) of 4.49 (95% CI: 3.47–5.51), while the type of restoration had an OR of 5.857 (95% CI: 5.125–6.589), indicating that these factors had the strongest influence on tooth survival. The model explained 37.6% of the variation in treatment outcomes.

According to the success criteria, 81% of cases were deemed successful. Success was significantly associated with several treatment-related factors, including preoperative PAI score (*p* = 0.001), follow-up time (*p* = 0.018), coronal restoration assessment and provider, type of restorative material, and post use/type (*p* < 0.0001). These associations are detailed in Table 1.

Multivariate logistic regression analysis identified preoperative PAI score and coronal restoration quality as significant predictors of endodontic treatment success. Higher PAI scores, inadequate RCT filling density, and inadequate coronal restorations were associated with an increased risk of treatment failure. Specifically, PAI scores of 3 and 4 had odds ratios of 3.802 (95% CI: 1.714–8.434) and 6.801 (95% CI: 2.612–17.710), respectively, compared with a PAI score of 1. Adequate coronal restoration served as a protective factor, with an odds ratio of 0.292 (95% CI: 0.105–0.811). The model explained 33.8% of the variance (Nagelkerke R^2^ = 0.338), indicating a moderate overall fit. Other factors, such as the presence of a post (*p* = 0.999) and treatment provider (*p* > 0.05), did not significantly affect treatment outcomes. (Table 2)

## 4. Discussion

The current study explored factors affecting the outcomes of initial and secondary RCT, in terms of both success and survival, within a patient population treated at an educational hospital. Consistent with previous studies from various regions, RCT demonstrated high success and survival rates, with an approximate 10% discrepancy between the two. While the overall success rate was 81%, tooth survival after RCT approached 90%, aligning with findings from the existing literature [6,13,18,30,31,32]. It is worth noting, however, that the majority of the teeth in the present study were followed for only 12–24 months.

Despite the significant association between clinical and radiographic success (*p* < 0.001), notable discrepancies were observed. In 42% of cases, radiographic failure was evident despite the absence of percussion pain, whereas 32% of cases exhibited clinical symptoms without corresponding radiographic signs of disease. Similar findings have been reported by da Rocha et al. (2022), emphasizing that while radiographic assessment is essential for evaluating RCT quality, it does not necessarily confirm the complete elimination of pathogenic biofilm [33]. Therefore, both radiographic and clinical evaluations are indispensable for accurately assessing RCT outcomes [15,31,32,33,34]. Radiographic failures without symptoms present the clinician with a management dilemma. The persistence of a radiographic lesion indicates that the underlying infection has not been completely resolved. Understanding the factors contributing to this condition and implementing appropriate management strategies are essential. Regular monitoring and determining the causes of failure are important steps in decision-making and in addressing this challenge and preventing potential complications [35].

Although patient-related factors such as age, medical status, periodontal health, oral hygiene, and oral habits showed potential associations with ETT survival, the regression model did not identify them as significant predictors. This finding is consistent with other studies examining the influence of patient age, gender, and periodontal health [20,31,33,34,36,37,38]. According to the multivariate regression model, the primary prognostic factors for survival were root canal filling density and the type and quality of coronal coverage. The influence of general health on treatment outcomes could not be fully determined in the present study due to the small proportion of patients with systemic conditions.

While patient-related factors were not statistically significant predictors in the regression model, the limited number of patients with a healthy periodontium (8.9%) suggests that periodontal status may have acted as a confounding factor. Compromised periodontal can negatively influence tooth prognosis by reducing load distribution and structural stability, even when endodontic healing occurs. Studies have demonstrated that the presence of moderate to severe attachment loss or furcation involvement is associated with lower survival rates of endodontically treated teeth [39,40].

Furthermore, excessive occlusal loading or parafunctional habits may exacerbate the risk of post-treatment complication. Chang et al. [41] reported that teeth under heavy occlusal stress or lacking cuspal protection exhibited significantly higher rates of vertical root fracture and coronal leakage. These findings underscore the multifactorial nature of endodontic prognosis, where both biological and biomechanical factors interact to influence long-term outcomes. Therefore, comprehensive assessment and management of occlusal forces and periodontal health are essential for optimizing post-treatment success and tooth longevity.

Regarding treatment success, multivariate logistic regression analysis identified preoperative PAI score, root canal filling density, and the quality of coronal coverage as significant prognostic factors. The presence and size of a periapical lesion were negatively associated with endodontic success, consistent with previous research [13,20,39,42,43,44].

It is important to note that the follow-up period in this study was less than 36 months for 78% of cases. This limited follow-up duration may have influenced the findings, as longer observation periods could potentially yield different results [6,13,32].

A well-condensed root canal filling increased the likelihood of both survival and success by 15%, consistent with previous studies [39,45,46]. Conversely, and in contrast to earlier reports [13,45,47], root canal filling length was not significantly associated with success. A possible explanation for this discrepancy is the relatively low proportion of inadequate treatments at recall, combined with the study’s low recall rate (16%), which is consistent with earlier findings [47,48]. This may have introduced selection bias that influenced the observed outcomes.

The finding that adequate endodontic filling density improves ETT survival and success is consistent with previous studies [13,37,46], which emphasize the importance of well-condensed three-dimensional root fillings in promoting periapical healing. In this study, sealer extrusion was not associated with non-healing, a result that contradicts a systematic review reporting a moderate risk of non-healing with sealer extrusion [49]. However, this observation aligns with more recent investigations of RCT outcomes [15,32,33].

The quality of coronal restoration had a strong impact on RCT outcomes, with adequate coronal coverage associated with nearly a 30% increase in both success and survival rates. Most studies support the role of coronal seal quality in ETT survival and success [15,32,37,42,46,47,50,51]. However, an earlier study conducted in undergraduate clinics reported that the condition of the coronal restoration did not influence RCT outcomes, although that study did not assess the quality of the RCT itself [33].

The role of restoration type as a prognostic factor remains controversial. In the regression model for RCT success, restoration type was not identified as a significant predictor, consistent with findings from several previous studies [39,46]. Although higher success rates have been reported in teeth with permanent restorations compared with temporary ones, the difference was not statistically significant. Likewise, no significant variation was observed between direct and indirect restorations [25,43,47,50].

Tooth survival, defined as the absence of clinical symptoms, was significantly associated with coronal restoration quality and the presence of an indirect restoration. Teeth restored with crowns were three times more likely to survive, a finding consistent with studies reporting that indirect restorations enhance ETT survival [18,39,52,53]. Although some short-term follow-up studies have not confirmed this association [50], the present study adds to the growing body of evidence supporting the benefits of indirect restorations in improving survival outcomes.

Post placement, on the other hand, was not significantly associated with either survival or success (*p* = 0.999), consistent with recent studies on ETT outcomes [15,46,54]. This finding contrasts with earlier reports suggesting that post placement may provide minor structural support benefits [6,55]. Differences in study samples, variations in post selection, and potential confounding factors may explain these discrepancies.

In the present study, the treatment provider did not influence success or survival, consistent with previous findings [1,37,46]. Conducted in an educational hospital with a favorable teacher-to-student ratio (1:4), the study ensured close supervision by experienced endodontists. Other investigations have reported variable success rates for RCT performed by undergraduate students, with some studies documenting lower rates of 60.7% and 75.5% [33,56]. These outcomes were assessed using strict criteria but did not account for the quality of endodontic treatment provided by students, which is a key factor influencing success.

The educational setting of this study provides insight into how supervision and operator experience influence RCT outcomes. Consistent with previous work [55], our results suggest that under structured supervision, students can achieve outcomes comparable to specialists. Da Rocha et al. (2022) [33] emphasized the importance of faculty calibration, standardized clinical protocols, and close monitoring in ensuring successful student-performed RCTs. A recent systematic review further confirmed that RCTs performed in undergraduate educational clinics can achieve success rates similar to those of experienced clinicians, largely due to rigorous supervision and evidence-based practice [35].

While studies by Fonzar et al. 2022 and Mareschi et al. 2020 reported slightly higher success rates in specialist settings, these differences may reflect case selection and access to advanced equipment rather than operator skill alone [39,57]. In the current study, the favorable supervision ratio and structured supervision may have contributed to the high survival and success rates observed. These findings underscore the importance of structured clinical training and quality assurance in dental education. Implementing continuous outcome monitoring, periodic radiographic audits, and structured feedback can sustain high treatment standards and support evidence-based learning. Moreover, linking student performance to patient outcomes can enhance accountability and promote long-term competency, reinforcing patient trust in educational hospitals. Continued investment in faculty calibration, training resources, and post-treatment follow-up will further strengthen both educational and clinical outcomes.

The present study has several limitations. First, it relied on the availability and accuracy of existing data. The low recall rate may have contributed to an overestimation of the reported success rate. It may have introduced selection bias, that affects the generalizability of the findings, as the participants who attended the recall visits may differ from those who did not. Additionally, the use of two-dimensional radiographic evaluation may not fully capture treatment outcomes. Incorporating three-dimensional cone-beam computed tomography (CBCT) could enhance accuracy and assessment precision [3].

Another potential source of bias is that most patients attending the follow-up visit had received treatment within the preceding 12–24 months. Patients treated earlier were less likely to return, which could have artificially inflated the reported success and survival rates. For example, individuals whose teeth had been extracted and replaced with implants would be less inclined to participate in the follow-up, leading to an underrepresentation of treatment failures.

Finally, the Nagelkerke R^2^ values indicate a moderate explanatory power of the models and suggesting that other unmeasured clinical or patient-level factors could also contribute to long-term outcomes. This study did not account for several treatment-related factors, including obturation technique, instrumentation technique, apical preparation size, and number of visits [46,47]. Future research should explore these factors in greater detail to determine their influence on RCT outcomes.

Despite these limitations, the findings provide valuable insights into the key determinants of endodontic success and align with a substantial body of literature emphasizing the importance of high-quality endodontic and restorative procedures. A major strength of this study is its integration of clinical data, offering a more comprehensive understanding of treatment outcomes by incorporating both clinical signs and symptoms assessed at follow-up. Long-term follow-up with combined clinical and radiographic evaluations remain essential to identify treatment failures and their underlying causes, thereby enabling more informed recommendations for future care.

## 5. Conclusions

This study underscores the complex interplay of factors that drive the survival and success of ETT. Three key elements emerge as critical determinants of treatment outcomes. First, the biological component represented by the extent of periapical pathology. Second, the treatment aspect, defined by the technical quality of the root canal filling. This plays a crucial role in ensuring effective disinfection and sealing of the root canal system. Third, restorative integrity, which provides a secure coronal, prevents microbial reinfection, safeguarding the tooth from future complications. Together, these elements form a triad that ultimately determines the fate of ETT, highlighting the importance of a comprehensive and high-quality approach to endodontic care.

## Figures and Tables

**Table 1:** Factors influencing the success of endodontically treated teeth according to the Chi-square test.

Influencing Factors		Success N = 374 (%)	Failure N = 87 (%)	Total N = 461 (%)	***p*** **Value**
Follow-up Time	12–24 months (median: 16.4 months)	205 (82.66)	43 (17.33)	248 (53.79)	
	25–36 months (median: 25 months)	90 (81.08)	21 (18.92)	111 (24)	
	37–48 months (median: 37 months)	29 (82.86)	6 (17.14)	35 (7.59)	
	49–60 months (median: 57 months)	32 (65.31)	17 (34.69)	49 (10.62)	
	61–73 months (median: 61 months)	18 (100.00)	0 (0.00)	18 (3.9)	
Treatment provider	Undergraduate student	178 (82.49)	38 (17.51)	216 (46.85)	0.8427
	Intern/GP	41 (77.36)	12 (22.64)	53 (11.49)	
	Endodontist	98 (80.99)	23 (19)	121 (26.24)	
	Board resident	57 (80.28)	14 (19.72)	71 (15.4)	
Periapical preoperative status	1	201 (87.07)	30 (12.93)	231 (50.11)	0.0010 *
	2	78 (81.25)	18 (18.75)	96 (20.82)	
	3	36 (75.00)	12 (25.00)	48 (10.41)	
	4	43 (71.67)	16 (28.33)	59 (12.79)	
	5	16 (59.26)	11 (40.74)	27 (5.85)	
RCT filling length	Adequate	218 (83.85)	41 (16.15)	259 (56.18)	0.1097
	Adequate with extruded sealer	144 (78.38)	40 (21.62)	184 (39.91)	
	Extruded	7 (77.78)	2 (22.22)	9 (1.94)	
	Short	5 (55.56)	4 (44.44)	9 (1.94)	
RCT filling density	Adequate	368 (81.46)	83 (18.54)	451 (97.83)	0.0631
	Inadequate apical third	4 (80.00)	1 (20.00)	5 (1.08)	
	Inadequate middle/coronal third	2 (40.00)	3 (60.00)	5 (1.08)	
Restoration material	Permanent	240 (86.33)	38 (13.67)	278 (60.3)	<0.0001 *
	Interim	134 (78.03)	37 (21.97)	171 (37.0)	
	Lost tooth	0 (0.00)	12 (100.00)	12 (2.6)	
Restoration material type	GIC	130 (78.44)	35 (21.56)	165 (35.79)	<0.0001 *
	Resin composite	29 (72.50)	11 (27.50)	40 (8.67)	
	Indirect restoration/Crown	215 (88.11)	29 (11.89)	244 (52.92)	
Coronal restoration provider	Undergraduate student	173 (83.25)	35 (16.75)	208 (45.11)	<0.0001 *
	Dental intern/GP	56 (84.85)	10 (15.15)	66 (14.31)	
	Endodontist	32 (80.00)	7 (20.00)	39 (8.46)	
	Board resident	61 (81.33)	14 (18.67)	75 (16.26)	
	Operative dentist/Prosthodontist	51 (85.00)	9 (15.00)	60 (13)	
	Lost tooth	1 (7.69)	12 (92.31)	13 (2.82)	
Restoration status assessment	Broken/lost	19 (52.78)	17 (47.22)	36 (7.80)	<0.0001 *
	Adequate	311 (84.78)	55 (15.22)	366 (79.39)	
	inadequate	44 (74.58)	15 (25.42)	59 (12.79)	
Post	Yes	212 (87.97)	29 (12.03)	241 (52.27)	<0.0001 *
	No	162 (77.62)	46 (22.38)	208 (45.11)	
	Lost tooth	0 (0.00)	12 (100.00)	12 (2.6)	
Post type	Fiber post	135 (89.40)	16 (10.60)	151 (32.75)	<0.0001 *
	Cast post	80 (86.02)	13 (13.98)	93 (20.17)	
	Lost tooth	0 (0.00)	12 (100.00)	12 (2.6)	
	No post	159 (77.29)	46 (22.71)	205 (44.47)	
Pulp diagnosis	Irreversible	109 (79.14)	29 (20.86)	138 (29.93)	0.7383
	Necrotic/Previously initiated	122 (80.79)	28 (19.21)	150 (32.52)	
	Reversible/Normal	39 (86.67)	6 (13.33)	45 (9.76)	
	Previously treated	104 (81.25)	24 (18.75)	128 (27.76)	
Periapical diagnosis	Symptomatic Apical Periodontitis	191 (79.92)	48 (20.08)	239 (51.84)	0.2583
	Asymptomatic Apical Periodontitis	118 (83.80)	23 (16.20)	141 (30.58)	
	Normal	52 (83.87)	10 (16.13)	62 (13.45)	
	Acute Apical Abscess	2 (50.00)	2 (50.00)	4 (0.86)	
	Chronic Apical Abscess	11 (68.75)	4 (31.25)	15 (3.25)	

* *p* ≤ 0.05 is considered statistically significant.

**Table 2:** Multivariate logistic regression analysis showing predictors for success of endodontically treated teeth.

Follow-Up	Sig.	Exp (B)	95% CI. for Exp (B)	
			Lower	Upper
	0.998	292,825,981.6	0.000	.
Treatment provider	0.627	1.505	0.290	7.819
Periapical Index pre-op status	0.001 *	3.802	1.714	8.434
RCT filling length	0.514	1.219	0.672	2.213
RCT filling density	0.020 *	9.410	1.418	62.428
Restoration material type	0.686	0.767	0.212	2.777
Coronal restoration provider	0.284	1.897	0.588	6.125
Restoration status assessment	0.018 *	0.292	0.105	0.811
Post	0.999	0.000	0.000	.
Post type	0.696	1.194	0.490	2.909

Level of significance: * *p* ≤ 0.05 is considered statistically significant.

## Data Availability

The datasets generated and analyzed during this study are available from the corresponding author upon reasonable request.

## Abbreviations

The following abbreviations are used in this manuscript:

ETT	Endodontically treated teeth
RCT	Root canal treatment
PAI	Periapical index
PNU	Princess Nourah Bint Abdulrahman University
CBCT	Cone-Beam Computed Tomography
